# Serum and peritoneal biomarkers for the early prediction of symptomatic anastomotic leakage in patients following laparoscopic low anterior resection: A single‐center prospective cohort study

**DOI:** 10.1002/cnr2.1781

**Published:** 2023-01-31

**Authors:** Xin‐Yu Qi, Fei Tan, Mao‐Xing Liu, Kai Xu, Pin Gao, Zhen‐Dan Yao, Nan Zhang, Hong Yang, Cheng‐Hai Zhang, Jia‐Di Xing, Ming Cui, Xiang‐Qian Su

**Affiliations:** ^1^ Department of Gastrointestinal Surgery IV Key Laboratory of Carcinogenesis and Translational Research (Ministry of Education), Peking University Cancer Hospital and Institute Beijing People's Republic of China

**Keywords:** anastomotic leakage, biomarkers, early prediction, nomogram, rectal cancer, symptomatic

## Abstract

**Background:**

Anastomotic leakage (AL) is one of the common complications after rectal cancer surgery. This study aimed to evaluate the combination of biomarkers for the early prediction of symptomatic AL after surgery.

**Methods:**

A prospective cohort study evaluated the serum and peritoneal biomarkers of patients who underwent laparoscopic low anterior resection (Lap LAR) from November 1, 2021, to May 1, 2022. Multivariate‐penalized logistic regression was performed to explore the independent biomarker with a *P*‐value <.1, and receiver operating characteristic (ROC) curve was used to analyze the area under the curve (AUC), sensitivity, and specificity of the independent biomarkers. A predictive model for symptomatic AL was built based on the independent biomarkers and was visualized with a nomogram. The calibration curve with the concordance index (c‐index) was further applied to evaluate the efficacy of the predictive model.

**Results:**

A total of 157 patients were included in this study, and 7 (4.5%) were diagnosed with symptomatic AL. C‐reactive protein/album ratio (CAR) on postoperative day 1 and systemic immune‐inflammation index (SII) and peritoneal interleukin‐6 (IL‐6) on postoperative day 3 were proven to be independent predictors for the early prediction of symptomatic AL. The optimal cutoff values of CAR, SII, and peritoneal IL‐6 were 1.04, 916.99, and 26430.09 pg/ml, respectively. Finally, the nomogram, including these predictors, was established, and the c‐index of this nomogram was 0.812, indicating that the nomogram could be used for potential clinical reference.

**Conclusion:**

The combination of CAR, SII, and peritoneal IL‐6 might contribute to the early prediction of symptomatic AL in patients following Lap LAR. Given the limitations of this study and the emergence of other novel biomarkers, multicenter prospective studies are worthy of further exploration.

## 1 INTRODUCTION

Anastomotic leakage (AL) following laparoscopic low anterior resection (Lap LAR) remains a catastrophic complication because it results in higher rates of mortality,^1^ increased healthcare costs,^1^ and poorer oncology prognosis.^2^ Despite the use of staplers and minimally invasive techniques, postoperative AL could still occur, and the reported incidence rate varies from 1% to 20%^3^ Moreover, postoperative AL is always diagnosed 5–8 days after surgery; even the delayed symptom beyond 30 days has also been reported.^4^ However, to date, AL diagnosis mainly depends on computed tomography (CT) scan, as well as clinical and biochemical assessments, making its detection difficult at an early stage. Furthermore, Dulk et al.^5^ demonstrated that a 2.5‐day delay in detecting AL could increase mortality from 24% to 39%. Hence, early AL detection is of great significance.

Direct comparison of biomarkers in different studies is relatively difficult because of the heterogeneity of patients and lack of consistency in AL diagnosis. However, studies have successively reported the significance of neutrophil‐to‐lymphocyte ratio (NLR), C‐reactive protein (CRP), and procalcitonin (PCT) in early AL diagnosis.^6^, ^7^, ^8^ In fact, any single test and multiple cutoff values of these serum biomarkers can alter the clinical reliability of AL early diagnosis. Moreover, besides peripheral blood tests, the exploration of biomarkers in drainage fluid has attracted more attention in recent years. Decreased pH value and increased peritoneal levels of cytokines were shown to be associated with AL, and measurement of these biomarkers seems to be more significant.^9^ Similar positive results were also shown in *Escherichia coli* and *Enterococcus faecalis* of drainage fluid through the reverse transcription–polymerase chain reaction (RT–PCR).^10^ Moreover, a high‐quality systematic review proposed that combinations of peritoneal drainage fluid and systemic biomarkers showed improvement in the predictive accuracy of AL than a single biomarker.^11^

Therefore, based on the above deficiencies, this prospective study aims to improve the early prediction of symptomatic AL following Lap LAR by combining different types of biomarkers and further exploring the optimal cutoff values.

## 2 METHODS

### 2.1 Patients

This study based on the clinical evaluation of patients who were diagnosed with rectal cancer and performed Lap LAR during a specific study period. For the sample size calculation, according to the observational study in the real world, we investigated the historical data of our center in recent 2 years, and the total sample size of patients who meets the inclusion criteria is probably 300–320 per year. Therefore, the number of consecutive patients enrolled in this prospective study is expected to be 150–160 half year. Considering the dropout rate (2%), the number of patients enrolled is expected to be 153–163 half year.

The single‐center prospective cohort study has registered in the Chinese Clinical Trial Registry (ChiCTR2100052406) on October 24, 2021. From November 1, 2021, to May 1, 2022, 160 consecutive patients with pathologically proven primary rectal cancer underwent Lap LAR at the Department of Gastrointestinal Surgery IV, Peking University Cancer Hospital and Institute. Two patients diagnosed with pneumonia after surgery and one diagnosed with a small intestinal leak following reoperation surgery were excluded. Thus, 157 patients were included in the final analysis. The Medical Ethics Committee of the Peking University Cancer Hospital approved this study and all patients provided written informed consent before enrollment in the study.

### 2.2 Inclusion and exclusion criteria

The inclusion criteria were as follows: (1) patients aged over 18 and preoperative pathologically confirmed primary rectal cancer; (2) patients who underwent Lap LAR; (3) concurrent with or without a defunctioning stoma; (4) patients with serum and peritoneal biomarkers on postoperative days 1, 3, and 5; (5) patients diagnosed with symptomatic AL (Grades B and C); and (6) patients with available clinicopathological data. The exclusion criteria were as follows: (1) emergency surgery; (2) infectious or autoimmune disease before surgery; (3) no pelvic drainage tube was placed during surgery; and (4) patients with other postoperative complications such as small intestinal leak or infections, including pneumonia, urinary tract infection, or incision infections.

### 2.3 Surgical procedure and research variables

All procedures were performed by three senior gastrointestinal surgeons from the same group in our unit. End‐to‐end anastomosis was used for colorectal anastomosis using a circular stapler (ETHICONTM Circular Stapler CDH25A; Ethicon Endo‐Surgery LLC). One pelvic drain was routinely placed around the anastomosis. Two protective measures, including defunctioning stoma and transanal tube drainage, were selectively performed during surgery based on the surgeon's discretion. The research variables mainly included two types of indicators, including serum and peritoneal biomarkers; the former included white blood cells (WBC), neutrophils (N), lymphocytes (L), monocytes (M), platelets (PLT), lymphocyte/monocyte ratio (LMR), platelets/lymphocytes ratio (PLR), prealbumin (PAlb), NLR, PCT, CRP, album (ALB), C‐reactive protein/album ratio (CAR), lymphocyte to C‐reactive protein ratio (LCR), prognostic nutritional index (PNI), and systemic immune‐inflammation index (SII). The calculations of PNI and SII were performed using the fomula: PNI = ALB (g/L) + 5 × L (10^9^/L); SII = PLT × NLR. The latter incorporated peritoneal interleukin‐1β (IL‐1β), interleukin‐6 (IL‐6), interleukin‐10 (IL‐10), interleukin‐8 (IL‐8), interleukin‐17A (IL‐17A), tumor necrosis factor‐α (TNF‐α), interferon‐γ (IFN‐γ), and pH value.

### 2.4 Definition

To ensure the accuracy of AL diagnosis, AL was defined as a defect at the anastomotic site with communication between the intra‐ and extraluminal compartments based on the International Study Group of Rectal Cancer recommendations of 2010.^12^ According to clinical symptoms and the AL severity, AL can be divided into symptomatic (Grades B and C) and asymptomatic (grade A). Therefore, in this study, AL diagnosis mainly includes clinical symptoms, serum and peritoneal biomarkers, and image examination. If patients had clinical symptoms, including fever, abdominal pain, and peritonitis, accompanied by elevated biomakers, the abdominal and pelvic CT was used for further diagnosis. Transanal X‐ray imaging was eventually required when the diagnosis was still uncertain.

### 2.5 Management of symptomatic AL

The management of symptomatic AL mainly includes two aspects: (1) conservative treatment, including fasting, parenteral nutrition support, antibiotics, and percutaneous catheter drainage, and (2) surgical treatment, including irrigation and drainage, as well as a defunctioning stoma.

### 2.6 Sample collection and storage

The serum biomarkers were measured by peripheral venous blood in the clinical laboratory of our hospital. All specimens were collected as fresh material within 1 h after regularly emptying the drainage ball in the morning on the first, third, and fifth postoperative days. The drainage fluid was collected using a sterile dispensing syringe for single use and deposited in a 2‐ml K_2_ ethylenediaminetetraacetic acid (EDTA) tube. The K_2_ EDTA tube was immediately sent to the basic laboratory, and the specimens were centrifuged (at 3000 rpm) for 15 min at 4°C. Afterward, the supernatant was separated and stored at −80°C until analysis.

### 2.7 Peritoneal biomarker analysis

The specimens were thawed, vortexed, and centrifuged (at 1000 rpm) at room temperature for 1 min before analysis. Specimens of cytokines were quantified using the cytokine combination assay kits (Celgene Biotech Co. Ltd., Jiangxi, China) upon the CytoFLEX machine according to the manufacturer's instructions. The pH values of the specimens were measured using a pH meter. Given that there are no normal reference intervals for cytokines and pH values in pelvic drainage, the normal reference ranges were determined by the non‐AL group in this study.

### 2.8 Statistical analysis

Based on whether the data are normally distributed, quantitative variables were expressed as mean ± standard deviation or median ± interquartile range (IQR) and were further analyzed using the two independent sample t‐test or Mann–Whitney U test. Categorical variables were expressed as percentages and compared using the Chi‐square test or Fisher's exact test. A two‐sided *P*‐value of <.05 was considered to indicate statistical significance. The multivariate‐penalized logistic regression was performed to explore the independent serum and peritoneal biomarkers on postoperative day 1, 3 and 5 with a *P*‐value of <.1 after surgery. Receiver operating characteristic (ROC) curve and area under the curve (AUC) were used to analyze independent biomarkers selected by multivariate‐penalized logistic regression. The AUC value represents the accuracy of the biomarker, 0.51–0.60 (fail), 0.61–0.70 (poor), 0.71–0.80 (fair), 0.81–0.90 (good), and 0.91–1.00 (excellent). Optimal cutoff values of variables were screened according to the Youden index; a *P*‐value of <.05 was considered to indicate statistical significance. Furthermore, the predictive model was established and shown as the nomogram based on the results of multivariate‐penalized logistic regression. The model performance was evaluated using Harrell's concordance index (c‐index). A c‐index of >0.75 is recognized as a clinically applicable value.^13^ The calibration curve with the c‐index was further applied to evaluate the performance of the prediction model. All statistical analyses were performed using the Statistical Package for the Social Sciences v.19.0 (IBM Corp., Armonk, NY, USA) and the Matching package in R, v.3.3.1 (R Foundation).

## 3 RESULTS

### 3.1 Study population

Among the 157 patients enrolled in this study, 7 (4.5%) were diagnosed with symptomatic AL. Among them, six patients were classified as Grade C and underwent a defunctioning stoma, whereas one patient with concurrent defunctioning stoma during radical surgery was evaluated as Grade B and treated with conservative treatment. The median time of symptomatic AL was 5 (IQR, 4–6) days after surgery, and no inhospital mortality occurred in our unit. The median follow‐up time was 28 (IQR, 26–30) days after surgery.

Clinicopathological data of patients with and without symptomatic AL are shown in Table 1. Although no statistical significance was found (*P* = .615), the incidence of symptomatic AL in male patients (85.7%) was six times that of female patients (14.3%). Patients with symptomatic AL had a longer median prolonged duration of operation than those without symptomatic AL (202 min vs. 161 min, *P* = .008). Moreover, patients diagnosed with symptomatic AL had a significantly closer median distance from the anal verge (6.5 vs. 12 cm, *P* = .010) and larger median tumor size (5.3 vs. 3.8 cm, *P* = .010) than those without symptomatic AL. Other clinicopathological variables, including age, body mass index, American Society of Anesthesiologists category, history of smoking, alcohol consumption and abdominal surgery, preoperative intestinal obstruction, hypertension, diabetes, neo‐adjuvant therapy, intraoperative blood loss and transfusion, combined organ resection, defunctioning stoma, transanal tube drainage, tumor differentiation, and pathological TNM category, were not obvious differences between the two groups in this study.

**TABLE 1:** Clinicopathological data of patients with and without symptomatic AL

Variables	Total no. of patients	Non‐AL	AL	P value
Gender (%)				.615
Male	110	104 (69.3)	6 (85.7)	
Female	47	46 (30.7)	1 (14.3)	
Age (median ± IQR) (years)		62 (56–65)	62 (59–70)	.434
Body mass index (median ± IQR) kg/m^2^		24.42 (22.94–26.45)	25.39 (24.31–26.73)	.323
ASA category (%)				.239
I	6	6 (4)	0 (0)	
II	143	137 (91.3)	6 (85.7)	
III	8	7 (4.7)	1 (14.3)	
Smoking (%)				.741
No	88	85 (56.7)	3 (42.9)	
Yes	69	65 (43.3)	4 (57.1)	
Alcohol consumption (%)				1.000
No	128	122 (81.3)	6 (85.7)	
Yes	29	28 (18.7)	1 (14.3)	
Previous history of abdominal surgery (%)				.566
No	134	127 (84.7)	7 (100)	
Yes	23	23 (15.3)	0 (0)	
Preoperative intestinal obstruction (%)				.566
No	134	127 (84.7)	7 (100)	
Yes	23	23 (15.3)	0 (0)	
Hypertension (%)				.125
No	99	97 (64.7)	2 (28.6)	
Yes	58	53 (35.3)	5 (71.4)	
Diabetes (%)				.496
No	118	114 (76)	4 (57.1)	
Yes	39	36 (24)	3 (42.9)	
Neoadjuvant therapy (%)				.329
No	124	120 (80)	4 (57.1)	
Yes	33	30 (20)	3 (42.9)	
Duration of operation (median ± IQR) (min)		161 (130–190)	202 (180–212)	.008*
Intraoperative blood loss (median ± IQR) (ml)		20 (20–30)	30 (20–50)	.137
Combined organ resection (%)				1.000
No	148	141 (94)	7 (100)	
Yes	9	9 (6)	0 (0)	
Intraoperative blood transfusion (%)				1.000
No	155	148 (98.7)	7 (100)	
Yes	2	2 (1.3)	0 (0)	
Defunctioning stoma (%)				.460
No	103	97 (64.7)	6 (85.7)	
Yes	54	53 (35.3)	1 (14.3)	
Transanal tube drainage (%)				.168
No	140	135 (90)	5 (71.4)	
Yes	17	15 (10)	2 (28.6)	
Distance from the anal verge (median ± IQR) (cm)		12 (10–15)	6.5 (6–12)	.010*
Size of tumor (median ± IQR) (cm)		3.8 (3–4.5)	5.3 (4.2–5.5)	.010*
Tumor differentiation (%)				.613
Well	15	15 (10.6)	0 (0)	
Moderate	122	116 (81.7)	6 (85.7)	
Poor	4	3 (2.1)	1 (14.3)	
Moderate‐poor	8	8 (5.6)	0 (0)	
Pathological tumor (T) category (%)				.896
T0	7	7 (4.7)	0 (0)	
T1	8	8 (5.3)	0 (0)	
T2	21	19 (12.7)	2 (28.6)	
T3	101	96 (64)	5 (71.4)	
T4	20	20 (13.3)	0 (0)	
Pathological node (N) category (%)				.964
N0	71	68 (45.3)	3 (42.9)	
N1	60	57 (38)	3 (42.9)	
N2	26	25 (16.7)	1 (14.3)	
Metastasis (%)				1.000
M0	127	121 (80.7)	6 (85.7)	
M1	30	29 (19.3)	1 (14.3)	

Abbreviation: AL, anastomotic leakage; ASA, American society of anesthesiologists; IQR, interquartile range.

*Note*: * represented *p* < .05.

### 3.2 Serum biomarkers

**FIGURE 1:**
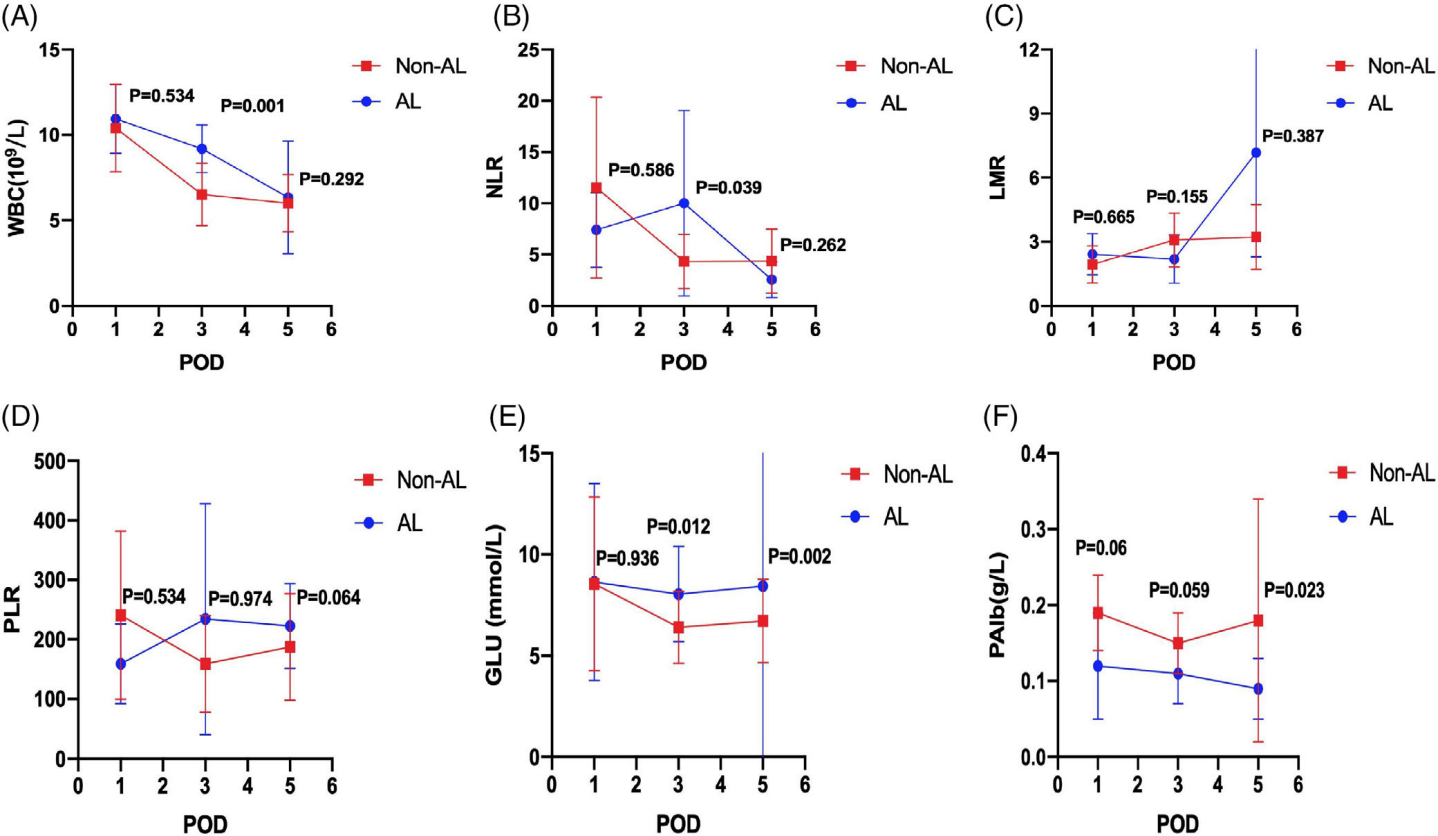
Mean with standard deviation of white blood cells (WBC) (A, 10^9^/L), NLR (B), LMR (C), PLR (D), GLU (E, mmol/L), and PAlb (F, g/L) on patients with AL and non‐AL on POD 1, 3 and 5.

**FIGURE 2:**
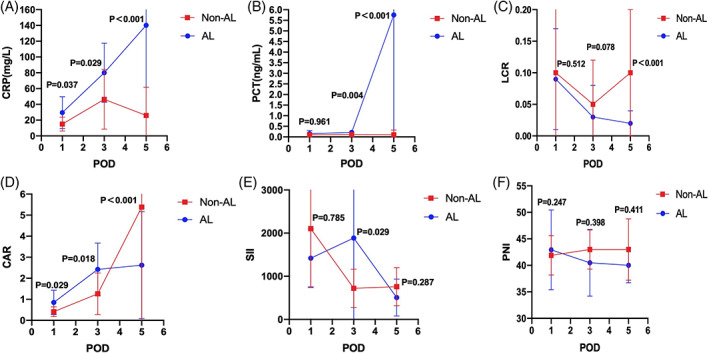
Mean with standard deviation of C‐reactive protein (CRP) (A, mg/L), PCT (B, ng/ml), LCR (C), CAR (D), SII (E), and PNI (F) on patients with AL and non‐AL on POD 1, 3 and 5.

There was no statistically significant difference in serum biomarkers between the two groups before surgery (Supplement Table 2). For postoperative serum biomarkers, patients with symptomatic AL had elevated mean CRP (29.48 vs. 15.02 mg/L, *P* = .037) and CAR (0.85 vs. 0.41, *P* = .029) than those without symptomatic AL on postoperative day 1 (POD 1) (Supplement Table 3). On POD 3, the median of WBC (8.96 vs. 6.29, *P* = .001), N (7.69 vs. 4.41, *P* = .001), NLR (5.98 vs. 3.55, *P* = .039), PCT (0.21 vs. 0.09 ng/ml, *P* = .004), GLU (8.61 vs. 5.92 mmoL/L, *P* = .012), and SII (978.60 vs. 618.71, *P* = .029), as well as CRP (85 vs. 40.15 mg/L, *P* = .029) and CAR (2.39 vs. 1.09, *P* = .018), were up to statistically significant differences in patients with symptomatic AL than those without symptomatic AL (Supplement Table 4). On POD 5, patients who developed symptomatic AL had greater median CRP (67.3 vs.16 mg/L, *P* = .000), CAR (1.67 vs. 0.41, *P* = .000), PCT (1.99 vs. 0.06 ng/ml, *P* = .000), GLU (8.63 vs. 6.22 mmoL/L, *P* = .002), and LCR (0.13 vs. 0.07, *P* = .000) than those without (Supplement Table 5). Moreover, the median of PAlb (0.07 vs. 0.16 g/L, *P* = .023) was lower in patients with symptomatic AL than in those without on POD 5. The graphs of the serum biomarkers are shown in Figures 1 and 2.

### 3.3 Peritoneal biomarkers

**FIGURE 3:**
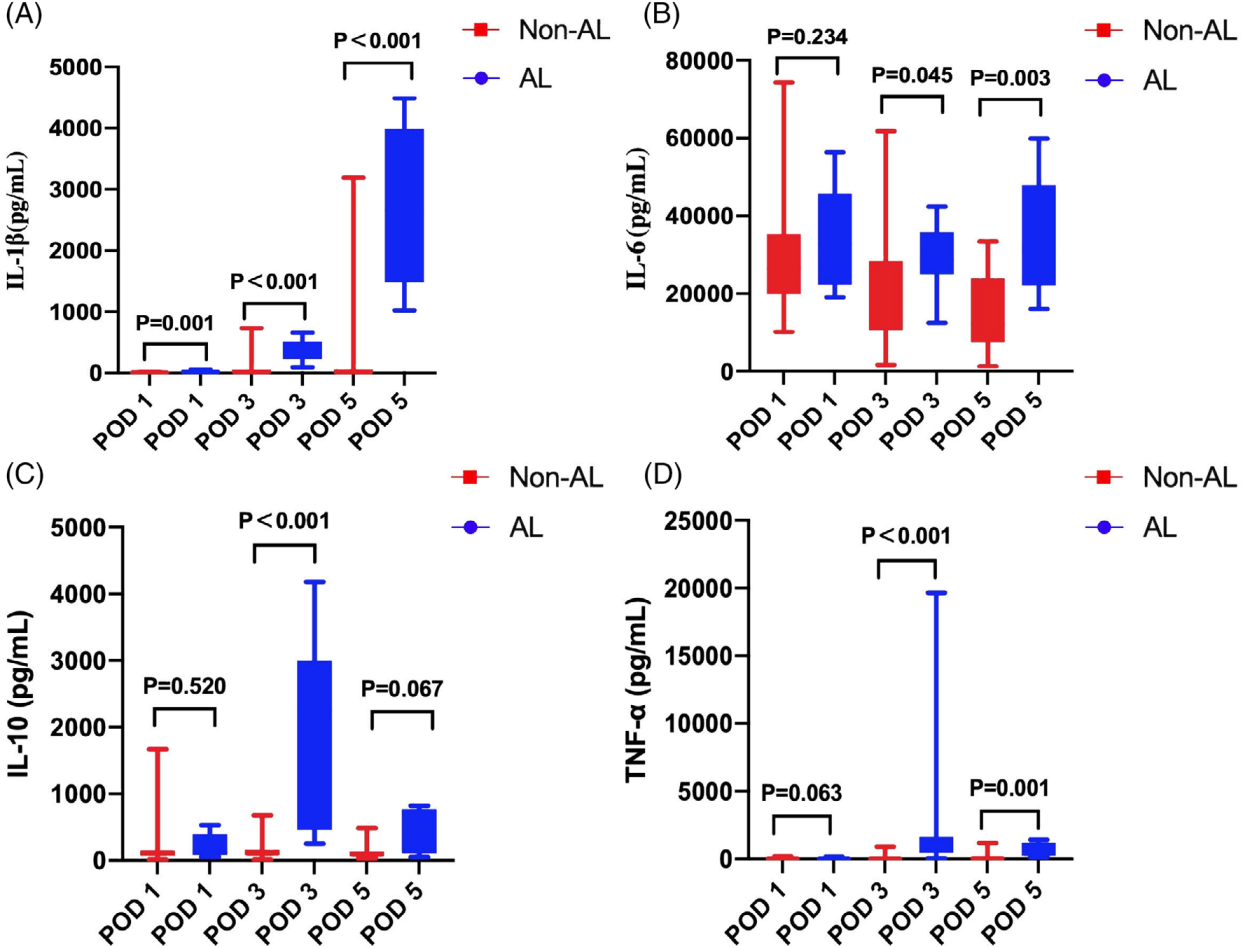
Median with interquartile range of peritoneal IL‐1β (A, pg/ml), IL‐6 (B, pg/ml), IL‐10 (C, pg/ml), and TNF‐α (D, pg/ml) on patients with AL and non‐AL on POD 1, 3 and 5.

**FIGURE 4:**
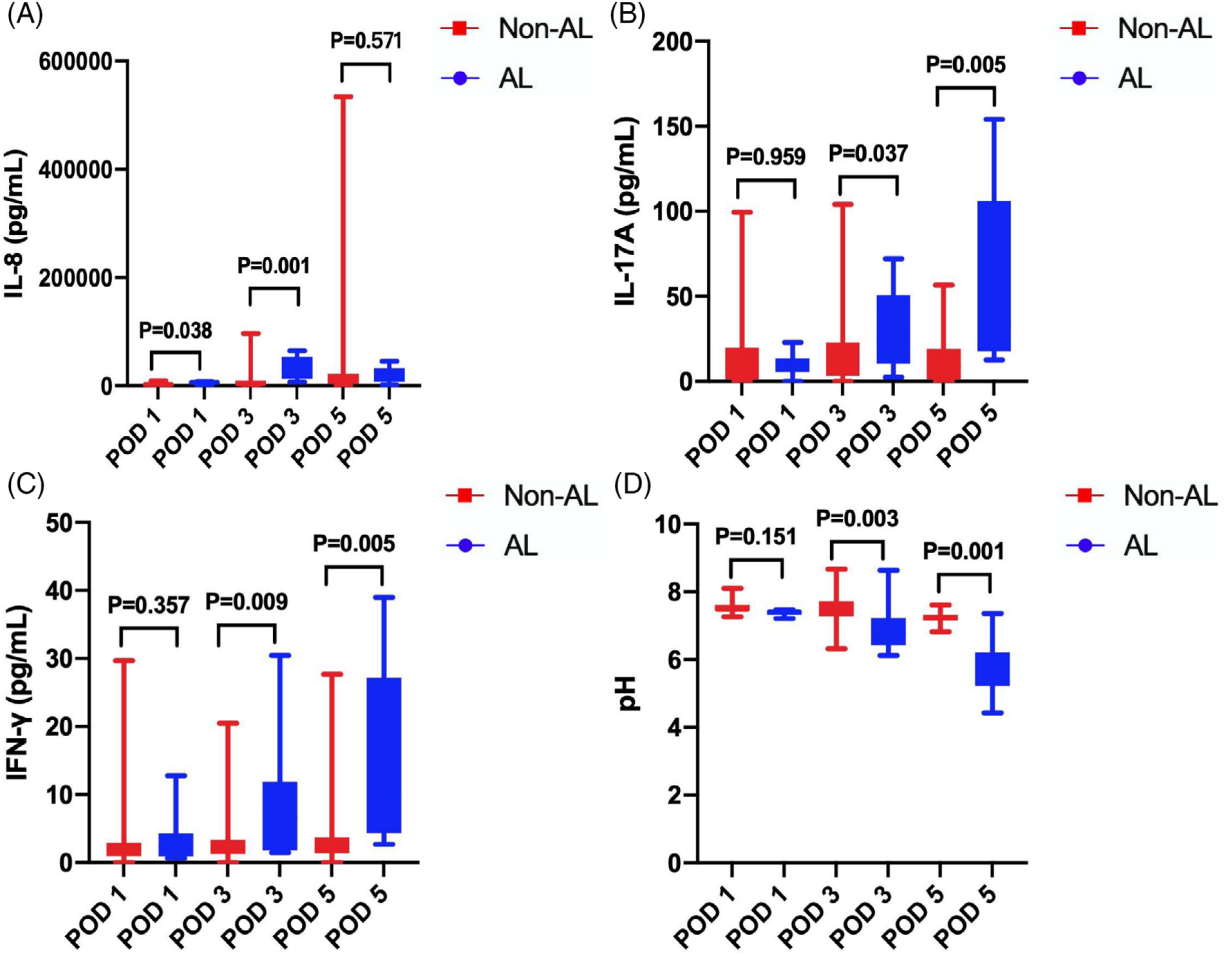
Median with interquartile range of peritoneal IL‐8 (A, pg/ml), IL‐17A (B, pg/ml), IFN‐γ (C, pg/ml), and pH (D) on patients with AL and non‐AL on POD 1, 3 and 5.

The peritoneal cytokine and pH values in both groups on 1, 3, and 5 days after surgery are shown in Supplement Tables 3, 4, and 5, respectively. The levels of IL‐1β in the symptomatic AL group promptly increased on postoperative days 1, 3, and 5 (*P* = .001; *P* = .000, and *P* = .000, respectively). IL‐6 and TNF‐α levels in the symptomatic AL group increased on POD 3 and 5 (*P* = .045 and *P* = .003, respectively), (*P* = .000 and *P* = .001, respectively). Similarly, increased IL‐17A and IFN‐γ levels were presented in the symptomatic AL group on POD 3 and 5 (*P* = .037 and *P* = .005, respectively), (*P* = .009 and *P* = .005, respectively). Moreover, IL‐10 levels were high in the symptomatic AL group on POD 3 (*P* = .000), whereas IL‐8 levels increased on POD 1 and 3 (*P* = .038 and *P* = .001, respectively). Compared with the non‐AL group, the median pH value was lower in the symptomatic AL group, with statistical significance on POD 3 and 5 (*P* = .003 and *P* = .001, respectively). The graphs of the peritoneal biomarkers are shown in Figures 3 and 4.

### 3.4 Multivariate‐penalized logistic regression and ROC curve analysis

**FIGURE 5:**
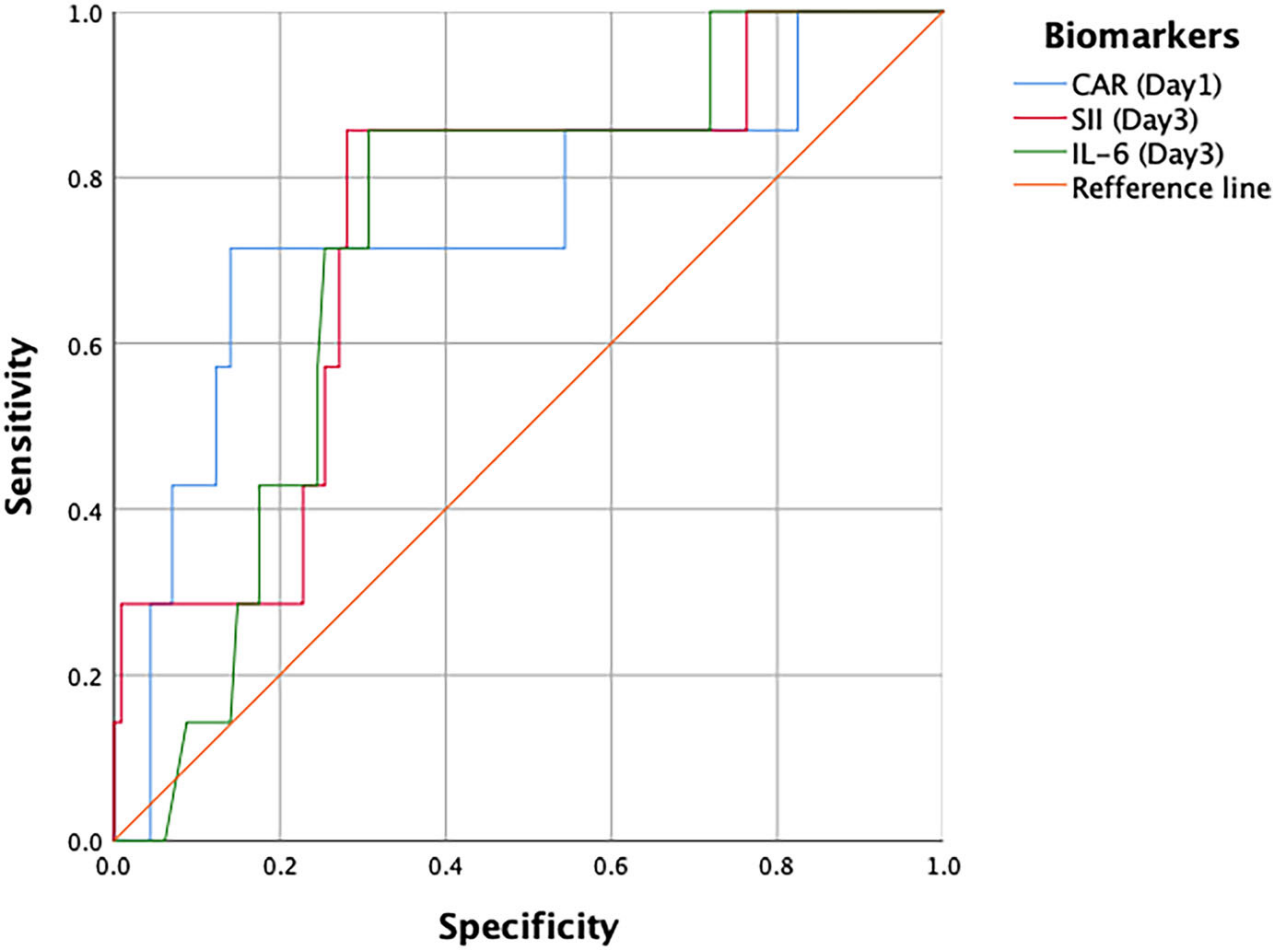
Receiver operating characteristic (ROC) curve for independent biomarkers for the early prediction of symptomatic AL.

After multivariate‐penalized logistic regression analyses, CAR (*P* = .085, OR 3.576 [95% CI, 0.746–15.997]) on POD 1 and SII (*P* = .035, OR 1.079 [95% CI, 1.008–1.170]) and peritoneal IL‐6 (*P* = .079, OR 3.442 [95% CI, 0.894–15.904]) on POD 3 were proven as independent predictors for symptomatic AL (Table 2). Furthermore, this study performed ROC curve analysis for the three independent biomarkers between the two groups (Table 3, Figure 5). The optimal cutoff value CAR at 1.04 showed the best AUC (0.744) with sensitivity and specificity of 71.4% and 57.4%, respectively. The optimal cutoff value of SII was 916.99 (AUC, 0.742; sensitivity, 85.7%; specificity, 57.6%; *P* = .032) and that of peritoneal IL‐6 was 26430.09 pg/ml (AUC, 0.726; sensitivity, 85.7%; specificity, 55%; *P* = .045). Among those biomarkers which were tested in different time after surgery, results obtained from 95% samples were CAR, SII and peritoneal IL‐6. Through further comparison, there were no definited association among the best cutoff values and mean or median of these three biomarkers in this study.

**TABLE 2:** Biomarkers of multivariate penalized logistic regression for symptomatic AL

Variables	Odds ratio	95% CI	*P* value
CAR (Day 1)	3.576	0.746–15.997	.085^†^
SII (Day 3)	1.079	1.008–1.170	.035^†^
IL‐6 (Day 3 pg/ml)	3.442	0.894–15.904	.079^†^

Abbreviations: AL anastomotic leakage; CAR, C‐reactive protein to albumin ratio; CI confidence interval; IL, interleukin; SII, systemic immune‐inflammation index.

*Note*: ^†^ *p* < .10.

**TABLE 3:** Receiver operating characteristic curve of biomarkers for symptomatic AL

Parameters	AUC	95% CI	*P* value	Cutoff	Sensitivity	Specificity
CAR (Day 1)	0.744	0.530–0.959	.030 *	1.04	71.4	57.4
SII (Day 3)	0.742	0.602–0.948	.032 *	916.99	85.7	57.6
IL‐6 (Day 3 pg/ml)	0.726	0.569–0.883	.045 *	26430.09	85.7	55.0

Abbreviations: AUC, area under the curve; CAR, C‐reactive protein to albumin ratio; CI, confidence interval; IL, interleukin; SII, systemic immune‐inflammation index; .

*Note*: * represented *p* < .05.

### 3.5 Nomogram of the prediction model

**FIGURE 6:**
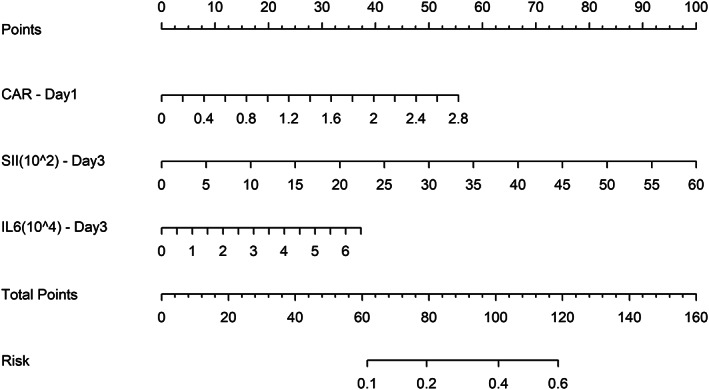
Nomogram of the predictive model for the early prediction of symptomatic AL.

**FIGURE 7:**
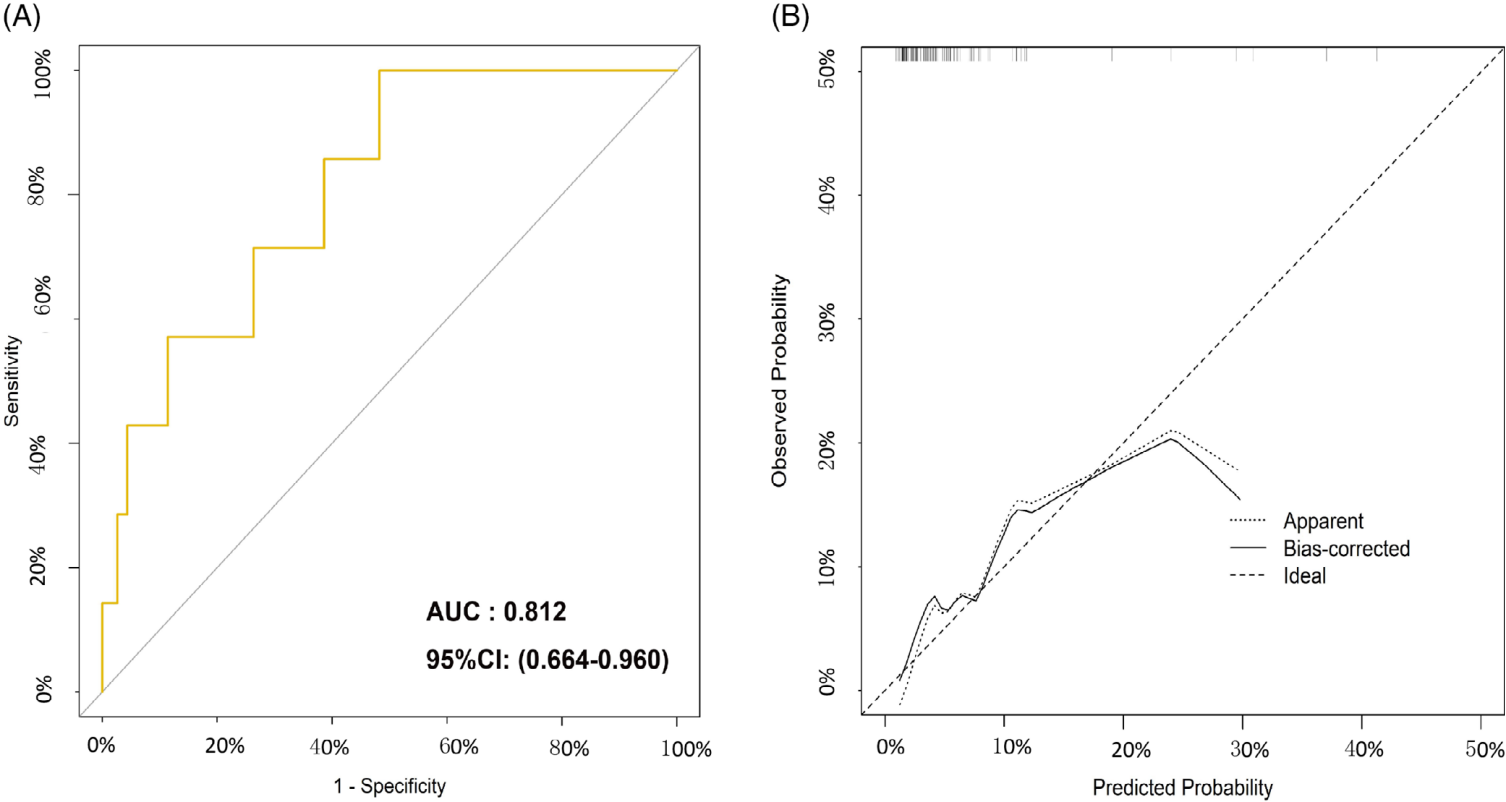
The performance (A) and calibration (B) curve of the nomogram of the predictive model

For the predictive model of biomarkers in symptomatic AL, the nomogram was established by means of combinations of the CAR on POD 1 and SII and peritoneal IL‐6 on POD 3 (Figure 6). The c‐index of this nomogram was 0.812 (95% CI, 0.664–0.960), indicating that this model had a good predictive efficacy for symptomatic AL between the two groups (Figure 7A). To evaluate the performance of the model, the calibration curve was further applied in this study (Figure 7B). The calibration curve indicated better consistency between the predicated and observed ends of this model.

## 4 DISCUSSION

With the application of the laparoscopic approach and enhanced recovery after colorectal surgery, the average postoperative hospital stay with no complications could be reduced to 5.5 days compared with those with complications.^8^ Unfortunately, AL after rectal surgery also occurs during this period, and AL of partially discharged patients might not be identified timely. Currently, anticipating when the AL will occur is still difficult; however, early AL detection is crucial for improving patient prognosis. This prospective study demonstrated that CAR on POD 1 and SII and peritoneal IL‐6 on POD 3 are independent predictors for symptomatic AL following Lap LAR. Furthermore, the nomogram showed that the predictive model of these independent biomarkers distributed a higher probability to patients with symptomatic AL than those without under 81.2% of the time. The optimal cutoff values for CAR, SII, and peritoneal IL‐6 were 1.04, 916.99, and 26430.09 pg/ml, respectively.

Several studies have reported serum CRP as an early predictor of AL after colorectal surgery.^7^, ^14^, ^15^ A recent prospective multicenter study involving 833 patients further demonstrated that a change in CRP level of more than 50 mg/L between any 2 consecutive days after surgery had a sensitivity of 85% in predicting AL.^16^ However, the use of CRP in early AL diagnosis remains controversial, with two main limitations. One is that the accuracy of diagnosis was restricted due to onther bacterial infections, and the other is the lack of definitive cutoff values. Therefore, CAR, a novel combination biomarker, has gradually entered the field of surgeons' vision and has been assessed in several studies. Ge et al.^17^ discovered that CAR has higher diagnostic accuracy than CRP alone for postoperative complications after colorectal surgery. However, the study did not reach a conclusion regarding the association between AL and CAR.^17^ Yu et al.^18^ successively reported that preoperative CAR and duration of operation were independent AL predictors among elderly patients after radical colorectal surgery. A recent study by Paliogiannis et al.^19^ further demonstrated that significantly elevated CAR was found in patients with AL than in those without AL, and a cutoff value of CAR >46 on postoperative day 4 showed higher accuracy in AL detection, with a sensitivity of 79.6% and specificity of 87.2%. In this study, although statistically significant differences in CAR at 1, 3, and 5 days after surgery were discovered, only the CAR on POD 1 was an independent predictor for symptomatic AL after multivariate‐penalized logistic regression. The AUC was 0.744, with a sensitivity of 71.7% and specificity of 57.4%. The reasons why the postoperative CAR was associated with symptomatic AL after surgery remain unclear. Serum albumin reflected the patient's nutritional status, and hypoalbuminemia severely impaired the healing ability of the anastomotic site due to decreased collagen synthesis.^20^ Serum CRP represents the severity of inflammation, and long‐lasting inflammation can impair collagen synthesis, ultimately leading to AL. More importantly, AL undoubtedly aggravated hypoalbuminemia and inflammation. Therefore, the intricate relationship between nutritional status, inflammation severity, and AL might contribute to explaining the possible reasons for CAR in the early prediction of symptomatic AL after rectal surgery. Meanwhile, the reasons why CAR has a higher predictive value than CRP or ALB alone in the early AL diagnosis are also stated here.

High SII levels have been reported to be associated with postoperative complications of oral cavity squamous cell carcinoma and ovarian peritoneal carcinomatosis.^21^, ^22^ Xie et al.^23^ first discovered that the combination of tumor markers and SII was a significant predictor of postoperative complications for patients after colorectal surgery. Based on the research by Xie,^23^ this study further demonstrated that patients with the cutoff value of SII > 916.99 were more proven to have symptomatic AL than those without. Since SII combines the characteristics of more peripheral blood inflammatory cells, it can comprehensively reflect the balance of a patient's systemic inflammation and immune status. When an inflammatory reaction occurs, peripheral blood neutrophils and platelets significantly increase, whereas nonspecific immune activation and damage lead to a decrease in lymphocytes, ultimately increasing the level of SII. Moreover, sepsis results from an imbalanced immune state and uncontrolled inflammation, and SII can be used to evaluate the severity and prognosis of sepsis to a certain degree.^24^ Furthermore, a series of studies have successively discovered that SII could predict the postoperative outcome and tumor progression of patients and is considered to be an effective prognostic factor for various malignant tumors, including nonsmall cell lung,^25^ hepatocellular,^26^ colorectal,^27^ and gastric cancers.^28^ Therefore, SII is of great significance in the prediction of postoperative complications and in tumor prognosis. In addition, the PNI, as another novel inflammatory composite index in recent years, did not show statistical differences and trends between the two groups, which might be due to the small sample size of this study, which is worthy of further exploration.

Growing high‐quality evidence show that cytokines in drainage fluid are essential for early AL detection after surgery.^29^, ^30^, ^31^, ^32^ The main reason for this is that the local cytokine concentration released from the surgical site increased faster than in peripheral blood within a few hours after surgery, as demonstrated in previous studies.^33^, ^34^ Although levels of all peritoneal cytokines in the symptomatic AL group were higher than those in the non‐AL group, in this study, only peritoneal IL‐6 was shown to be an independent predictor after multivariate‐penalized logistic regression. The results were consistent with the prospective study by Yamamoto et al.^32^ who demonstrated that levels of peritoneal IL‐6 were obviously higher in patients with peritonitis on POD 3. The second largest multicenter prospective study by Sammour et al.^29^ reported that peritoneal IL‐6 is helpful for early postoperative AL diagnosis on POD 1, whereas the first largest multicenter prospective study by Sparreboom et al.^35^ found that peritoneal IL‐6 has no significance in early postoperative AL detection on the first 3 days following rectal cancer resection. The possible reasons for this opposite result are mainly inconsistent definitions of AL and differences in inclusion criteria after careful analysis. Moreover, the updated high‐quality meta‐analysis^36^ of eight studies further confirmed that peritoneal IL‐6 levels could be used as a diagnostic marker for AL after colorectal surgery. In response to surgical stress, proinflammatory cytokines, including IL‐6 and TNF‐α, released from macrophages could produce an inflammatory reaction. This process was inhibited by anti‐inflammatory cytokines, such as IL‐10.^37^ Moreover, an experimental animal study has demonstrated that systemic IL‐6 was harmful to the healing of colonic anastomoses of rats.^38^ Therefore, an imbalance of this process may be an adverse event of AL in theory. Furthermore, the values of measured peritoneal IL‐6 were ten times higher than TNF‐α, with the longer half‐life of IL‐6, making the measurement of IL‐6 more clinically application.^39^ Given that the cutoff values of peritoneal IL‐6 widely vary, more studies are needed to explore the optimal cutoff. This study also attempted to investigate the roles of peritoneal pH, IL‐17A, and IFN‐γ in detecting symptomatic AL, but statistical significance was not reached following multivariate analysis.

In the past decade, researchers have extensively explored possible biomarkers of early postoperative AL diagnosis to improve the prognosis of patients with AL. A prospective study^40^ reported that increased peritoneal lactate concentration has higher sensitivity and specificity than clinical scoring systems for AL cases. In a recent systematic review, matrix metalloprotease (MMP), a novel biomarker of tissue repair, has been shown to be associated with AL development.^41^ A multicenter prospective study by Komen et al.^42^ suggested that quantitative PCR for *E. faecalis* on POD 3 could be a quick screening tool for symptomatic AL. Furthermore, the concept that the diagnostic efficacy of any single biomarker type is difficult to use for early AL identification has been clinically accepted. A multicenter cohort study by Sparreboom et al.^35^ first proposed that the combination of serum CRP and MMP‐9 showed the best model performance (c‐index = 0.78) on POD 3. The AUC values of any independent predictor in this study hint at a fair result for diagnostic efficacy, but combinations of these biomarkers showed a good result for early symptomatic postoperative AL prediction. Therefore, the authors believe that combining more types of biomarkers will aid in the early prediction of symptomatic AL in the future. Nevertheless, we must realize that these biomarkers are powerful auxiliary indicators, not the gold standard for AL in any situation.

This study has limitations that should be considered. First, although the internal validation of this prediction model performed well, the efficacy of external validation needs to be further verified in clinical trials. Second, using combination assay kits to measure cytokines was inexpensive and convenient. Given the inherent problems of cytokines, the measurement of peritoneal cytokines should be immediately performed in the laboratory. Unfortunately, limitations of laboratory process still need to be considered. Last, the small sample size and single‐center characteristic of this study might bring bias in data analysis, which we have acknowledged.

## 5 CONCLUSIONS

Conclusively, this prospective study firstly demonstrated that combinations of CAR, SII, and peritoneal IL‐6 might contribute to the early prediction of symptomatic AL in patients following Lap LAR, especially on POD 3. In view of the limitations of this study and the emergence of other novel biomarkers, multicenter prospective studies are worthy of further exploration in the future.

## AUTHOR CONTRIBUTIONS

**Xin‐Yu Qi:** Data curation (supporting); formal analysis (supporting); methodology (supporting); software (supporting); writing – original draft (lead); writing – review and editing (equal). **Fei Tan:** Validation (supporting); visualization (supporting); writing – original draft (equal); writing – review and editing (lead). **Maoxing Liu:** Visualization (lead); writing – original draft (equal); writing – review and editing (equal). **Kai Xu:** Resources (equal); software (lead); supervision (equal). **Pin Gao:** Investigation (equal); methodology (lead); resources (equal). **Zhendan Yao:** Data curation (equal); formal analysis (equal); methodology (supporting). **Nan Zhang:** Investigation (lead); methodology (equal); project administration (equal). **Hong Yang:** Data curation (lead); formal analysis (equal); resources (equal). **Chenghai Zhang:** Data curation (equal); formal analysis (equal); investigation (supporting); project administration (lead). **Jiadi Xing:** Data curation (equal); formal analysis (lead); methodology (supporting); project administration (equal); supervision (equal). **MING Cui:** Conceptualization (equal); data curation (supporting); formal analysis (supporting); funding acquisition (equal); resources (lead). **Xiangqian Su:** Conceptualization (lead); data curation (supporting); formal analysis (supporting); funding acquisition (lead); project administration (supporting); resources (supporting); supervision (supporting).

## CONFLICT OF INTEREST

The authors have stated explicitly that there are no conflicts of interest in connection with this article.

## ETHICS STATEMENT

The study complies with the Declaration of Helsinki and was approved by the Ethics Committee of Peking University Cancer Hospital.

## Supporting information


**Supplement Table 2.** Comparison of biomarkers for patients with and without symptomatic AL before surgery

**Supplement Table 3.** Comparison of biomarkers for patients with and without symptomatic AL on postoperative day 1

**Supplement Table 4.** Comparison of biomarkers for patients with and without symptomatic AL on postoperative day 3

**Supplement Table 5.** Comparison of biomarkers for patients with and without symptomatic AL on postoperative day 5

## Data Availability

The datasets used and/or analyzed during the current study are available from the corresponding author on reasonable request.
