# Supplementary material for: Serum and peritoneal biomarkers for the early prediction of symptomatic anastomotic leakage in patients following laparoscopic low anterior resection: A single‐center prospective cohort study
Source: Cancer Rep (Hoboken). 2023 Jan 31;6(4):e1781. doi: 10.1002/cnr2.1781 (PMC10075299; doi:10.1002/cnr2.1781)
Supplement: Supplementary file 1 — Supplement Table 2. Comparison of biomarkers for patients with and without symptomatic AL before surgery [file CNR2-6-e1781-s001.docx]

Supplement Table 2. Comparison of biomarkers for patients with and without symptomatic AL before surgery

| Variables | Non-AL | AL | *P* value |
| --- | --- | --- | --- |
| WBC (median ± IQR) 10^9^/L | 6.49 (5.19 - 7.66 ) | 7.10 (5.77 - 8.23) | 0.438 |
| Neutrophils (median ± IQR) 10^9^/L | 4.29 (3.04 - 5.05) | 4.83 (3.17 - 5.50) | 0.548 |
| Lymphocytes (mean ± SD) 10^9^/L | 1.74 ± 0.69 | 1.86 ± 0.65 | 0.664 |
| Monocytes (median ± IQR) 10^9^/L | 0.37 (0.30 - 0.46) | 0.48 (0.40 - 0.52) | 0.079 |
| Platelets (median ± IQR) 10^9^/L | 213 (178.50 - 263.25) | 233 (214 - 268) | 0.194 |
| NLR (median ± IQR) 10^9^/L | 2.37 (1.80 - 3.60) | 2.71 (1.65 - 3.86) | 0.965 |
| LMR (mean ± SD) 10^9^/L | 4.57 (3.31 -5.62) | 3.43 (2.27 - 5.74) | 0.512 |
| PLR (median ± IQR) 10^9^/L | 131.48 (101.66 - 174.97 ) | 120.23 (96.06 - 190.86) | 0.887 |
| Hemoglobin (mean ± SD) g/L | 138.29 ± 18.47 | 145.14 ± 10.70 | 0.334 |
| ALB (mean ± SD) g/L | 45.15 ± 3.08 | 44.01 ± 3.03 | 0.346 |
| PNI (mean ± SD) | 53.68 (51 - 57.04) | 50.95 (50.30 -58.95) | 0.650 |
| SII (median ± IQR) | 530.43 (364.66 - 854.92) | 580.69 (366.53 - 1368.47) | 0.870 |

*Abbreviations: WBC, white blood cells; NLR, neutrophil to lymphocyte ratio; LMR, lymphocyte to monocyte ratio; PLR, platelet to lymphocyte ratio; ALB, album; PNI, prognostic nutritional index; SII, systemic immune-inflammation index; AL, anastomotic leakage; SD, standard deviation; IQR, interquartile range.*
