# Supplementary material for: Serum and peritoneal biomarkers for the early prediction of symptomatic anastomotic leakage in patients following laparoscopic low anterior resection: A single‐center prospective cohort study
Source: Cancer Rep (Hoboken). 2023 Jan 31;6(4):e1781. doi: 10.1002/cnr2.1781 (PMC10075299; doi:10.1002/cnr2.1781)
Supplement: Supplementary file 2 — Supplement Table 3. Comparison of biomarkers for patients with and without symptomatic AL on postoperative day 1 [file CNR2-6-e1781-s002.docx]

Supplement Table 3. Comparison of biomarkers for patients with and without symptomatic AL on postoperative day 1

| Variables | Non-AL | AL | *P* value |
| --- | --- | --- | --- |
| WBC (median ± IQR) 10^9^/L | 9.94 (8.79 - 12.06) | 10.48 (9.37 -12.75) | 0.323 |
| Neutrophils (mean ± SD) 10^9^/L | 8.78 ± 2.34 | 8.75 ± 2.10 | 0.606 |
| Lymphocytes (mean ± SD) 10^9^/L | 1.05 ± 0.52 | 1.52 ± 0.93 | 0.160 |
| Monocytes (median ± IQR) 10^9^/L | 0.51 (0.41 - 0.64) | 0.65 (0.42 - 0.82) | 0.471 |
| Platelets (median ± IQR) 10^9^/L | 179.5 (162 - 224.75) | 196 (178.50 - 216) | 0.249 |
| NLR (median ± IQR) 10^9^/L | 8.66 (4.99 -13.40) | 9.07 (3.69 -10.31) | 0.586 |
| LMR (mean ± SD) 10^9^/L | 1.93 ± 0.87 | 2.41 ± 0.97 | 0.665 |
| PLR (median ± IQR) 10^9^/L | 178.17 (128.49 - 336.63) | 179.09 ( 89.97 - 218.32) | 0.534 |
| PAlb (mean ± SD) g/L | 0.19 ± 0.05 | 0.12 ± 0.07 | 0.060 |
| PCT (median ± IQR) ng/mL | 0.06 (0.05 - 0.93) | 0.09 (0.06 - 0.27) | 0.961 |
| CRP (mean ± SD) mg/L | 15.02 ± 8.82 | 29.48 ± 20.25 | 0.037^†^ |
| ALB (median ± IQR) g/L | 36.90 (34.35 -37.83) | 36.4 (31.90 - 38.15) | 0.471 |
| CAR (mean ± SD) | 0.41 ± 0.23 | 0.85 ± 0.58 | 0.029^†^ |
| Glucose (median ± IQR) mmol/L | 7.00 (5.39 -10.35) | 6.98 (5.45 -12.65) | 0.936 |
| LCR (median ± IQR) | 0.73 (0.04 - 0.11) | 0.60 (0.03 - 0.17) | 0.512 |
| PNI (mean ± SD) | 41.88 ± 3.71 | 42.92 ± 7.52 | 0.247 |
| SII (median ± IQR) | 1714.29 (966.46 - 2919.50) | 1756.59 (715.48 -1952.73) | 0.785 |
| IL-1β (median ± IQR) pg/mL | 5.86 (3.03 -9.05) | 16.61 (9.74 - 29.12) | 0.001^†^ |
| IL-6 (mean ± SD) pg/mL | 30042.75 ± 10972.06 | 37555.81 ± 14161.88 | 0.234 |
| IL-10 (median ± IQR) pg/mL | 109.64 (75.75 - 132.97 ) | 122.8 (75.31 - 335.30) | 0.520 |
| TNF-α (median ± IQR) pg/mL | 4.77 (2.57 - 13.72) | 15.31 (8.62 - 121.18) | 0.063 |
| IL-8 (median ± IQR) pg/mL | 2057.58 (1329.25 - 3743.47) | 5609.47 (2670.74 - 7488.77) | 0.038^†^ |
| IL-17 (median ± IQR) pg/mL | 7.53 (2.35 - 12.24) | 8.62 (2.79 - 18.22) | 0.959 |
| IFN-γ (median ± IQR) pg/mL | 1.58 (0.94 - 2.03) | 2.35 (1.26 - 8.55) | 0.357 |
| pH (median ± IQR) | 7.50 (7.46 - 7.62) | 7.45 (7.42 - 7.54) | 0.151 |

*Abbreviations: WBC, white blood cells; NLR, neutrophil to lymphocyte ratio; LMR, lymphocyte to monocyte ratio; PLR, platelet to lymphocyte ratio; PAlb, prealbumin; PCT, procalcitonin; CRP, C-reactive protein; ALB, album; CAR, C-reactive protein to albumin ratio; LCR, lymphocyte to C-reactive protein ratio; PNI, prognostic nutritional index; SII, systemic immune-inflammation index; AL, anastomotic leakage; IL, interleukin; IFN, interferon; SD, standard deviation; IQR, interquartile range.*

*Note: ^†^p < 0.05.*
