# Supplementary material for: Serum and peritoneal biomarkers for the early prediction of symptomatic anastomotic leakage in patients following laparoscopic low anterior resection: A single‐center prospective cohort study
Source: Cancer Rep (Hoboken). 2023 Jan 31;6(4):e1781. doi: 10.1002/cnr2.1781 (PMC10075299; doi:10.1002/cnr2.1781)
Supplement: Supplementary file 3 — Supplement Table 4. Comparison of biomarkers for patients with and without symptomatic AL on postoperative day 3 [file CNR2-6-e1781-s004.docx]

Supplement Table 4. Comparison of biomarkers for patients with and without symptomatic AL on postoperative day 3

| Variables | Non-AL | AL | *P* value |
| --- | --- | --- | --- |
| WBC (median ± IQR) 10^9^/L | 6.29 (5.49 - 7.36) | 8.96 (8.50 - 10.71) | 0.001^†^ |
| Neutrophils (median ± IQR) 10^9^/L | 4.41 (3.67 - 5.15) | 7.69 (6.78 - 8.07) | 0.001^†^ |
| Lymphocytes (mean ± SD) 10^9^/L | 1.25 ± 0.46 | 1.12 ± 0.55 | 0.720 |
| Monocytes (median ± IQR) 10^9^/L | 0.41 (0.32 - 0.50) | 0.52 (0.47 - 0.66) | 0.048^†^ |
| Platelets (median ± IQR) 10^9^/L | 165.5 (142.50 - 198.25) | 174 (161 - 207) | 0.382 |
| NLR (median ± IQR) 10^9^/L | 3.55 (2.64 - 4.79) | 5.98 (5.62- 16.36) | 0.039^†^ |
| LMR (mean ± SD) 10^9^/L | 3.08 ± 1.26 | 2.18 ± 1.13 | 0.155 |
| PLR (median ± IQR) 10^9^/L | 143.15 (105.29 - 175.74) | 129.84 (123.40 - 397.87 ) | 0.974 |
| PAlb (median ± IQR) g/L | 0.14 (0.12 - 0.17) | 0.11 (0.06 - 0.14) | 0.059 |
| PCT (median ± IQR) ng/mL | 0.09 (0.06 - 0.13) | 0.21 (0.14 - 0.26) | 0.004^†^ |
| CRP (median ± IQR) mg/L | 40.15 (23.80 - 53.03) | 85 (64.5 - 108.20) | 0.029^†^ |
| ALB (median ± IQR) g/L | 36.3 (34.70 - 38.00) | 35.6 (30.20 - 37.80) | 0.281 |
| CAR (median ± IQR) | 1.09 (0.67 - 1.43) | 2.39 (1.75 - 3.58) | 0.018^†^ |
| Glucose (median ± IQR) mmol/L | 5.92 (5.23 - 7.14) | 8.61 (6.12 - 9.31) | 0.012^†^ |
| LCR (median ± IQR) | 0.04 (0.02 - 0.06) | 0.01 (0.00 - 0.03) | 0.078 |
| PNI (median ± IQR) | 42.35 (40.54 - 45.09) | 40.45 (33.75 - 46.45) | 0.398 |
| SII (median ± IQR) | 618.71 (435.68 - 860.27) | 978.60 (922.32 - 3059.64) | 0.029^†^ |
| IL-1β (median ± IQR) pg/mL | 15.85 (6.94 - 50.79) | 416.17 (227.06 - 510.58) | 0.000^†^ |
| IL-6 (median ± IQR) pg/mL | 19538.03 (12810.32 - 30972.27 ) | 28649.23 (24972.5 - 35865.46) | 0.045^†^ |
| IL-10 (median ± IQR) pg/mL | 110.26 (87.98 - 184.91) | 2243.08 (456.42 - 3002.35) | 0.000^†^ |
| TNF-α (median ± IQR) pg/mL | 8.43 (4.59 - 15.79) | 1013.35 (439.27 - 1625.32) | 0.000^†^ |
| IL-8 (median ± IQR) pg/mL | 3795.6 (1901.47 - 9618.09) | 32000 (13217.01 - 53425.87) | 0.001^†^ |
| IL-17 (median ± IQR) pg/mL | 9.60 (3.59 - 22.84) | 26.20 (10.49 - 50.67) | 0.037^†^ |
| IFN-γ (median ± IQR) pg/mL | 2.31 (1.25 - 3.32) | 6.67 (1.79 - 11.81) | 0.009^†^ |
| pH (median ± IQR) | 7.56 (7.33 - 7.82) | 6.76 (6.43 - 7.23) | 0.003^†^ |

*Abbreviations: WBC, white blood cells; NLR, neutrophil to lymphocyte ratio; LMR, lymphocyte to monocyte ratio; PLR, platelet to lymphocyte ratio; PAlb, prealbumin; PCT, procalcitonin; CRP, C-reactive protein; ALB, album; CAR, C-reactive protein to albumin ratio; LCR, lymphocyte to C-reactive protein ratio; PNI, prognostic nutritional index; SII, systemic immune-inflammation index; IL, interleukin; IFN, interferon; AL, anastomotic leakage; SD, standard deviation; IQR, interquartile range.*

*Note: ^†^p < 0.05.*
