# Supplementary material for: Serum and peritoneal biomarkers for the early prediction of symptomatic anastomotic leakage in patients following laparoscopic low anterior resection: A single‐center prospective cohort study
Source: Cancer Rep (Hoboken). 2023 Jan 31;6(4):e1781. doi: 10.1002/cnr2.1781 (PMC10075299; doi:10.1002/cnr2.1781)
Supplement: Supplementary file 4 — Supplement Table 5. Comparison of biomarkers for patients with and without symptomatic AL on postoperative day 5 [file CNR2-6-e1781-s003.docx]

Supplement Table 5. Comparison of biomarkers for patients with and without symptomatic AL on postoperative day 5

| Variables | Non-AL | AL | *P* value |
| --- | --- | --- | --- |
| WBC (median ± IQR) 10^9^/L | 5.72 (4.80 - 7.25) | 7.35 (1.85 - 8.66) | 0.292 |
| Neutrophils (median ± IQR) 10^9^/L | 3.89 (3.09 - 4.93) | 5.53 (1.04 - 6.88) | 0.184 |
| Lymphocytes (median ± IQR) 10^9^/L | 1.09 (0.76 - 1.53) | 0.84 (0.51 - 0.93) | 0.051 |
| Monocytes (median ± IQR) 10^9^/L | 0.38 (0.32 - 0.50) | 0.53 (0.07 - 0.61) | 0.333 |
| Platelets (median ± IQR) 10^9^/L | 179 (150 - 237) | 199 (146 - 227) | 0.737 |
| NLR (median ± IQR) 10^9^/L | 3.37 (2.57 - 5.13) | 2.04 (1.58 - 3.28) | 0.262 |
| LMR (median ± IQR) 10^9^/L | 3.02 (2.32 - 4.03) | 7.29 (4.76 - 9.64) | 0.387 |
| PLR (median ± IQR) 10^9^/L | 161.05 (131.08 - 226.17) | 236.91 (191.15 - 261.59) | 0.064 |
| PAlb (median ± IQR) g/L | 0.16 (0.13 - 0.19) | 0.07 (0.07 - 0.11) | 0.023^†^ |
| PCT (median ± IQR) ng/mL | 0.06 (0.04 - 0.08) | 1.99 (1.05 - 8.58) | 0.000^†^ |
| CRP (median ± IQR) mg/L | 16 (9.10 - 25.4) | 67.3 (55.9 - 188) | 0.000^†^ |
| ALB (median ± IQR) g/L | 37.5 (36.4 - 39.1) | 35.8 (34.2 - 36.55) | 0.559 |
| CAR (median ± IQR) | 0.41 (0.22 - 0.71) | 1.67 (1.24 - 2.52) | 0.000^†^ |
| Glucose (median ± IQR) mmol/L | 6.22 (5.45 - 7.13) | 8.63 (8.18 - 8.82) | 0.002^†^ |
| LCR (median ± IQR) | 0.07 (0.04 - 0.12) | 0.13 (0.01 - 0.02) | 0.000^†^ |
| PNI (median ± IQR) | 43.3 (40.15 - 45.15) | 39.85 (38.33 - 41.63) | 0.411 |
| SII (median ± IQR) | 580.11 (473.52 - 977.93) | 297.73 (260.21 - 549.02) | 0.287 |
| IL-1β (median ± IQR) pg/mL | 15.87 (6.31 - 48.47) | 3072.18 (1431.19 - 4237.73) | 0.000^†^ |
| IL-6 (median ± IQR) pg/mL | 13542.24 (7475.97 - 24713.81) | 33570.96 (28930.08 - 53896.20) | 0.003^†^ |
| IL-10 (median ± IQR) pg/mL | 93.15 (62.42 - 139.42) | 442.68 (104.27 - 772.74) | 0.067 |
| TNF-α (median ± IQR) pg/mL | 21.42 (11.25 - 57.29) | 913.20 (462.37 - 1320.95) | 0.001^†^ |
| IL-8 (median ± IQR) pg/mL | 11411.54 (3984.69 - 22291.47) | 17749.25 (13655.61 - 38622.20) | 0.571 |
| IL-17 (median ± IQR) pg/mL | 7.92 (1.19 - 16.65) | 45.24 (25.21 - 130.05) | 0.005^†^ |
| IFN-γ (median ± IQR) pg/mL | 2.19 (1.40 - 3.69) | 12.40 (6.89 - 33.06) | 0.005^†^ |
| pH (mean ± SD) | 7.23 (7.15 - 7.32) | 5.62 (5.23 - 6.21) | 0.001^†^ |

*Abbreviations: WBC, white blood cells; NLR, neutrophil to lymphocyte ratio; LMR, lymphocyte to monocyte ratio; PLR, platelet to lymphocyte ratio; PAlb, prealbumin; PCT, procalcitonin; CRP, C-reactive protein; ALB, album; CAR, C-reactive protein to albumin ratio; LCR, lymphocyte to C-reactive protein ratio; PNI, prognostic nutritional index; SII, systemic immune-inflammation index; IL, interleukin; IFN, interferon; AL, anastomotic leakage; SD, standard deviation; IQR, interquartile range.*

*Note: ^†^p < 0.05.*
